# Exosomal circSTRBP from cancer cells facilitates gastric cancer progression via regulating miR‐1294/miR‐593‐3p/E2F2 axis

**DOI:** 10.1111/jcmm.18217

**Published:** 2024-03-23

**Authors:** Yin Wang, Rong Zou, Deke Li, Xiankui Gao, Xingjun Lu

**Affiliations:** ^1^ Department of Gastroenterology Bozhou Hospital affiliated to Anhui Medical University Bozhou China; ^2^ Department of Gastroenterology, Wuhan Puren Hospital Wuhan University of Science and Technology Wuhan China; ^3^ Department of Anesthesiology The Fifth Hospital of Wuhan China

**Keywords:** circRNA, circSTRBP, E2F2 by modulating, exosome, gastric cancer, miRNA

## Abstract

CircRNAs represent a new class of non‐coding RNAs which show aberrant expression in diverse cancers, such as gastric cancer (GC). circSTRBP, for instance, is suggested to be overexpressed in GC cells and tissues. However, the biological role of circSTRBP in the progression of GC and the potential mechanisms have not been investigated. circSTRBP levels within GC cells and tissues were measured by RT‐qPCR. The stability of circSTRBP was assessed by actinomycin D and Ribonuclease R treatment. Cell proliferation, migration, invasion and in vitro angiogenic abilities after circSTRBP knockdown were analysed through CCK‐8 assay, transwell culture system and the tube formation assay. The interaction of circSTRBP with the predicted target microRNA (miRNA) was examined by RNA immunoprecipitation and luciferase reporter assays. Xenograft tumour model was established to evaluate the role of exosomal circSTRBP in the tumour formation of GC cells. circSTRBP was upregulated in GC cells and tissues, and there was an increased level of circSTRBP in GC‐derived exosomes. circSTRBP in the exosomes enhanced GC cell growth and migration in vitro, which modulates E2F Transcription Factor 2 (E2F2) expression through targeting miR‐1294 and miR‐593‐3p. Additionally, exosomal circSTRBP promoted the tumour growth of GC cells in the xenograft model. Exosomal circSTRBP is implicated in the progression of GC by modulating the activity of miR‐1294/miR‐593‐3p/E2F2 axis.

## 1 INTRODUCTION

Gastric cancer (GC) is ranked as the fifth most prevalent malignancies globally, with over 1 million new cases reported annually.^1^ The highest rate is reported in East and Central Asia, Latin America and Eastern Europe.^2^ Multiple risk factors are associated with the occurrence of GC, such as age, gender, infection with *Helicobacter pylori*, low vegetable consumption, high‐salt intake and tobacco smoking.^2^ Unfortunately, GC is asymptomatic at the early stage, and the patients gradually develop symptoms such as dyspepsia, anorexia, unintentional weight loss and abdominal pain with the disease progression. GC‐related mortality is high due to the high frequency of diagnosis at advanced stages, and the death in GC patients accounts for the third most cancer‐associated mortality worldwide.^1^ Endoscopic or surgical resection is the first‐line treatment for the primary and localized GC. GC patients with metastasis is difficult to treat, and systemic chemotherapy is generally applied to prolong the life span of the patients.^1^, ^3^

Advancement in molecular technology has enabled the identification of biomarkers for the diagnostic and prognostic purposes, as well as the biomarkers with the potential to inform treatment response and disease recurrence. Circular RNAs (circRNAs), for instance, have been extensively studied as potential cancer markers in recent years.^4^, ^5^ CircRNAs represent a novel class of functional non‐coding RNAs (ncRNAs), and over 100,000 unique circRNAs have been identified in the human transcriptome.^6^, ^7^ These ncRNAs are single‐stranded and covalently closed molecules generated from precursor mRNAs through the back‐splicing mechanism.^8^ Compared with their linear counterparts, circRNAs are highly stable with an extended half‐life. Different groups of circRNAs are often expressed within specific cells or tissues.^8^ In addition, circRNAs can be exported outside the cell through exosomes and are secreted by most cells into biofluids such as blood, urine and saliva.

Numerous studies have shown the biological significance of circRNAs during tumour genesis and development.^4^, ^5^ For example, circ_0009172 was reported to suppress GC progression by regulating miRNA‐485‐3p/NTRK3 axis.^9^ Circ‐ITCH acts as a tumour suppressor to inhibit the metastasis of GC by targeting miR‐199a‐5p/Klotho axis.^10^ In addition, circRAB31 functions as a molecular sponge for miR‐885‐5p to suppress the malignancy of GC cells via modulating PTEN/PI3K/AKT pathway.^11^ Nevertheless, the functional roles of most circRNAs in the malignant progression of GC remain unclear. Genome‐wide high‐throughput sequencing of circRNAs has revealed the profile of circRNA deregulation in GC patients.^12^ circSTRBP (also known as hsa_circRNA_104907 or hsa_circ_0001888), was found to be upregulated within GC cells and tissues.^13^ However, the functional engagement of circSTRBP in dictating the malignancy of GC cells and in the progression of GC has not yet been clarified. In our study, we explored the functional role of circSTRBP in regulating the malignant progression of GC using in vitro and in vivo experiments. We revealed that circSTRBP interacted with hsa‐miR‐1294 (a miRNA that was reported to be underexpressed in GC^14^, ^15^, ^16^) and hsa‐miR‐593‐3p. Both miRNAs could negatively regulate E2F Transcription Factor 2 (E2F2) expression, an oncogene involved in the development of GC.^17^ We further showed that circSTRBP was secreted in the exosomes by GC cells to promote cancer progression.

## 2 MATERIALS AND METHODS

### 2.1 Human GC tissues

A total of 80 pairs of GC tumour specimens and matched normal tissues were obtained by surgery from GC patients at Bozhou Hospital affiliated with the Anhui Medical University. Each sample was evaluated by two independent pathologists. This study gained approval from Bozhou Hospital affiliated with the Anhui Medical University Ethics Committee. All cases provided informed consent.

### 2.2 Cell culture and transfection

GC cells (HGC27, AGS, MKN‐45 and MKN74) together with healthy gastric epithelial GSE‐1 cells were acquired from biological cell bank of the Chinese Academy of Science (Shanghai, China). The cells were cultivated within RPMI‐1640 Medium (11875101, Invitrogen, Shanghai, China) with 10% fetal bovine serum under 37°C and 5% CO_2_ condition. Meanwhile, small interfering RNA (siRNA) of circSTRBP, inhibitors of miR‐1294 and miR‐593‐3p, and the negative controls were produced in Genomeditech Co., Ltd. (Shanghai, China). Lipofectamine 2000 (11668019, Invitrogen, Shanghai, China) was utilized in cell transfection experiment in line with specific instructions. Forty‐eight hours after the transfection, the cells were harvested for further functional assays.

### 2.3 Exosome isolation

Total Exosome Isolation Reagent (from serum) (4478360, Invitrogen, Shanghai, China) was adopted for isolating exosomes from cultured cells. 1 × 10^7^ HGC27 cells or GSE‐1 cells were cultured for 72 h, and the supernatant was collected and mixed with Total Exosome Isolation Reagent in 10:1 ratio (volume). The sample was mixed and subjected to 30‐min incubation under 4°C, followed by a 10‐min centrifugation at 10,000 *g* under ambient temperature. The exosomal pellet was reconstituted into 1 × PBS (400 μL) and preserved under −20°C prior to use.

### 2.4 Real‐time quantitative PCR (qRT‐PCR)

Total cellular and tissue RNAs were isolated from HGC‐27/AGS cells or tissues by TRIzol Reagent (15596026, Invitrogen, Shanghai, China) in line with specific protocols. PrimeScript II 1st Strand cDNA Synthesis Kit (6110A, TaKaRa, Tokyo, Japan) was employed for synthesizing cDNA from 1 μg of total RNA. Thereafter, by adopting SYBR Premix Ex Taq II (RR82WR, Takara, Tokyo, USA) together with corresponding primers, CFX96 Touch Real‐time PCR detection system (CFX96, Bio‐Rad, Hercules, CA, USA) was adopted for qRT‐PCR. 2^−ΔΔCT^ approach was utilized to determine miRNA and circRNA expression, with U6 as the internal reference and GAPDH as the endogenous control. Sequences of primers are shown below:

5′‐AAGAGCCCACGCTAACTTTGA‐3′ (forward [F]), 5′‐TCATAAGCCTGAGTCCCCTGA‐3′ (reverse [R]) for circSTRBP;

5′‐GGACCGAGTCAAGTCAAAGG‐3′ (F), 5′‐GGAGGCTGAGGCAGAAGAAT‐3′ (R) for circ_0001013; 5′‐GTGTCTCTGCTGGGGTT‐3′ (F), 5′‐CAGTGCGTGTCGTGGAGT‐3′ (R) for miR‐593‐3p; 5′‐GTGAGGTTGGCATTGTTG‐3′ (F), 5′‐CAGTGCGTGTCGTGGAGT‐3′ (R) for miR‐1294; 5′‐AGACCTTCTTGTATAAGCACTGT‐3′ (F), 5′‐CAGTGCGTGTCGTGGAGT‐3′ (R) for miR‐1248; New: 5′‐GCTTCGGCAGCACATATACTAAAAT‐3′ (F), 5′‐CGCTTCACGAATTTGCGTGTCAT‐3′ (R) for U6; and 5′‐TGTTCGTCATGGGTGTGAAC‐3 (F), 5′‐ATGGCATGGACTGTGGTCAT‐3 (R) for GAPDH.

### 2.5 RNase R digestion and Actinomycin D treatment

RNA (10 μg) isolated from AGS and HGC27 cells (5 × 10^4^ cells/well) was incubated under 37°C with/without 3 U/μg of RNase R for 20 min (R304332, Epicenter, Madison, WI, USA). The remaining RNA was subjected to the purification using RNeasy MinElute Cleaning Kit (74204, Qiagen, Stockholm, Sweden) and then analysed by qRT‐PCR. Alternatively, the RNA samples were treated with 2 μg/mL Actinomycin D (A1410, Sigma, Shanghai, China) to assess the stability of circSTRBP and its linear counterpart.

### 2.6 Western blot (WB) assay

RIPA buffer containing phosphatase and proteinase inhibitors (K10034, KeyGen, Beijing, China) was adopted for protein sample collection Thereafter, the Enhanced BCA Protein Assay Kit (P0010S, Beyotime, Beijing, China) was employed for protein concentration determination. Aliquots of proteins were separated through 10% SDS‐PAGE and transferred to PVDF membranes (IPVH15150, Millipore, Darmstadt, Germany). Thereafter, 5% bovine serum albumin (A9576, Sigma, Shanghai, China) was utilized to block the membranes for 1 hour, followed by the incubation with the target antibodies (Abcam, Cambridge, UK): anti‐E‐cadherin (1:1000; Abcam, ab15148), anti‐N‐cadherin (1:1000; Abcam, ab76011), anti‐VEGFA (1:1000; Abcam, ab46154) or anti‐β‐actin (1:1000; Abcam, ab8226) overnight under 4°C. Afterwards, HRP‐labelled secondary antibody (1:10,000; 61‐6520, Invitrogen, Shanghai, China) was utilized to further label the membranes for 1 h at room temperature. After signal development, protein blots were visualized and quantified by ImageJ Software Version 1.53t (NIH, Bethesda, MD, USA).

### 2.7 Immunofluorescence staining

After the extraction of cancer cell exosomes, the samples were conjugated by Dil (red) dye (72485, Sigma, Shanghai, China) before the addition into AGS and HGC‐27 cells overnight. Incubation. The location of Dil‐conjugated exosomes was observed under the Leica AM6000 microscope (Leica, Wetzlar, Germany).

### 2.8 Cell Counting Kit‐8 assay

CCK‐8 assay kit (HY‐K0301; MedChemExpress, Shanghai, China) was used for measuring cell growth in line with specific protocols. After transfection, cells were inoculated into the 96‐well plates for indicted culture periods. CCK‐8 reagent (20 μL) was added into every well and the plate was incubated for another 2‐h period under 37°C. The measurement of absorbance (OD) value was conducted using a microplate reader at 450 nm.

### 2.9 Transwell assay

HGC‐27 and AGS cells (5 × 10^4^) in serum‐free medium were inoculated into Matrigel‐coated top Transwell chambers for invasion assay (pore size, 8 μm; CLS‐CC‐027, Costar, San Diego, CA, USA). Migration assay was conducted in the plate without Matrigel coating. Complete medium (600 μL) containing 10% FBS was added into bottom chamber. After 48 h, cells with no invasion or migration were eliminated from the top chamber, while those on the lower surface were subjected to 30‐min fixation using 4% paraformaldehyde and 15‐min staining using 0.1% crystal violet (C0121; Beyotime, Beijing, China).

### 2.10 Tube formation assay

Cells (4 × 10^3^/well, Corning Glass Works) were plated into the Matrigel (356237, BD Bioscience, Los Angeles, CA, USA)‐coated 96‐well plates, followed by 8‐hour culture within DMEM that contained 10% FBS under 37°C and 5% CO_2_. The average number of capillary‐like branches was observed with a microscope (200×).

### 2.11 Fluorescence in situ hybridization (FISH)

HGC‐27 and AGS cells were fixed and permeabilized before hybridization. The permeabilized cells were hybridized with Cy‐3‐labelled circSTRBP probes (RiboBio, Guangzhou, China) for a 16‐h period under 37°C. The cells were then rinsed in saline‐sodium citrate buffer under 42°C for three times, followed by 30‐min incubation with Tween® supplemented within PBS that contained 5% BSA under ambient temperature. The Fluorescent In Situ Hybridization Kit (R1067, RiboBio, Guangzhou, China) was employed to detect the hybridization signals.

### 2.12 RNA pull‐down assay

Lipofectamine RNAiMAX Transfection Reagent (13778150, Invitrogen, Shanghai, China) was used to transfect NC probe (50 nM) or biotinylated miRNA mimic into AGS and HGC27 cells 48 h post‐transfection; the cells were harvested and lysed, followed by the incubation with C‐1 magnetic beads (65001, Life Technologies, Shanghai, China) for a 2‐h period under 4°C. TRIzol (15596026, Invitrogen, Shanghai, China) was used to extract the RNA sample for subsequent qRT‐PCR quantification.

### 2.13 Luciferase reporter analysis

The luciferase reporters containing wild‐type or mutated binding sites were prepared by the GenePhama (Shanghai, China). The reporters were delivered into HGC‐27 and AGS cells by Lipofectamine 2000 reagent (11668019, Invitrogen, Shanghai, China) together with miR‐NC or miRNA mimic. After 48 h, the Luciferase Reporter Gene Detection Kit (LUC1‐1KT, Sigma, Shanghai, China) was used for the determination of luciferase activities in line with specific protocols.

### 2.14 RNA immunoprecipitation

AGS and HGC27 cells were lysed with the cell lysis buffer in Magna RIP® RNA‐Binding Protein Immunoprecipitation Kit (17‐700, Millipore, Temecula, CA, USA) following specific instructions. The lysates were then incubated with RIP buffer containing magnetic beads conjugated with human anti‐Ago2 antibody (ab156870, Abcam) or nonspecific IgG antibody (ab37450, Abcam) for 18 h under 4°C. TRIzol (15596026, Invitrogen, Shanghai, China) was used to extract the immunoprecipitated RNAs, followed by qRT‐PCR detection.

### 2.15 Calcein acetoxymethyl ester/propidium iodide (calcein‐AM/PI) staining

To examine cell viability and cell death, a cell viability/death double staining kit (04511, Sigma, Shanghai, China) was used for simultaneous labelling of viable and dead cells. In brief, cells were washed with PBS and adjusted to the cell density at 1 × 10^5^ cells/mL. One hundred microlitres of assay solution was mixed with 1 mL of cell suspension for the staining at 37°C for 15 min. After the staining, the cells were viewed under a fluorescence microscope with 490 nm excitation laser to simultaneously monitor the viable and dead cells. Living cells produce green fluorescence due to the conversion of calcein acetoxymethyl ester (calcein‐AM) to calcein, while dead cells produce red fluorescence as a result of DNA staining by propidium iodide (PI).

### 2.16 Xenograft tumour model

AGS cells (1 × 10^7^) were suspended in 200 μL normal saline and subcutaneously injected into right flanks of BABL/c male nude mice (6‐8‐week‐old; Beijing Weitong Lihua Experimental Animal Technology Co., Ltd.). The mice were then randomly assigned to exo‐si‐circSTRBP or exo‐si‐NC group (*n* = 6/group). On Day 7 following tumour cell injection, each mouse was intratumorally injected with 10 μg of exosomes extracted from circSTRBP siRNA‐transfected AGS cells or AGS cells transfected with the control siRNA. All the mice were sacrificed by cervical dislocation on Day 28 and the tumour tissues were harvested for weight determination and subsequent analyses.

### 2.17 Statistical analysis

SPSS19.0 (IBM, New York, NY, USA) was employed for statistical analysis. Data were displayed as mean ± SD. Wilcoxon test or Student's *t*‐test was applied to compare continuous variable between two samples. The analyses of data from multiple groups were conducted by one‐way anova. Kaplan–Meier (KM) curve was plotted to analyse survival of cancer patients, and log‐rank *t*‐test was applied in the result analysis. *p < 0.05; **p < 0.01; ***p < 0.001 represent different degrees of statistical significance.

## 3 RESULTS

### 3.1 circSTRBP shows an elevated expression level in GC cells and tissues

**FIGURE 1:**
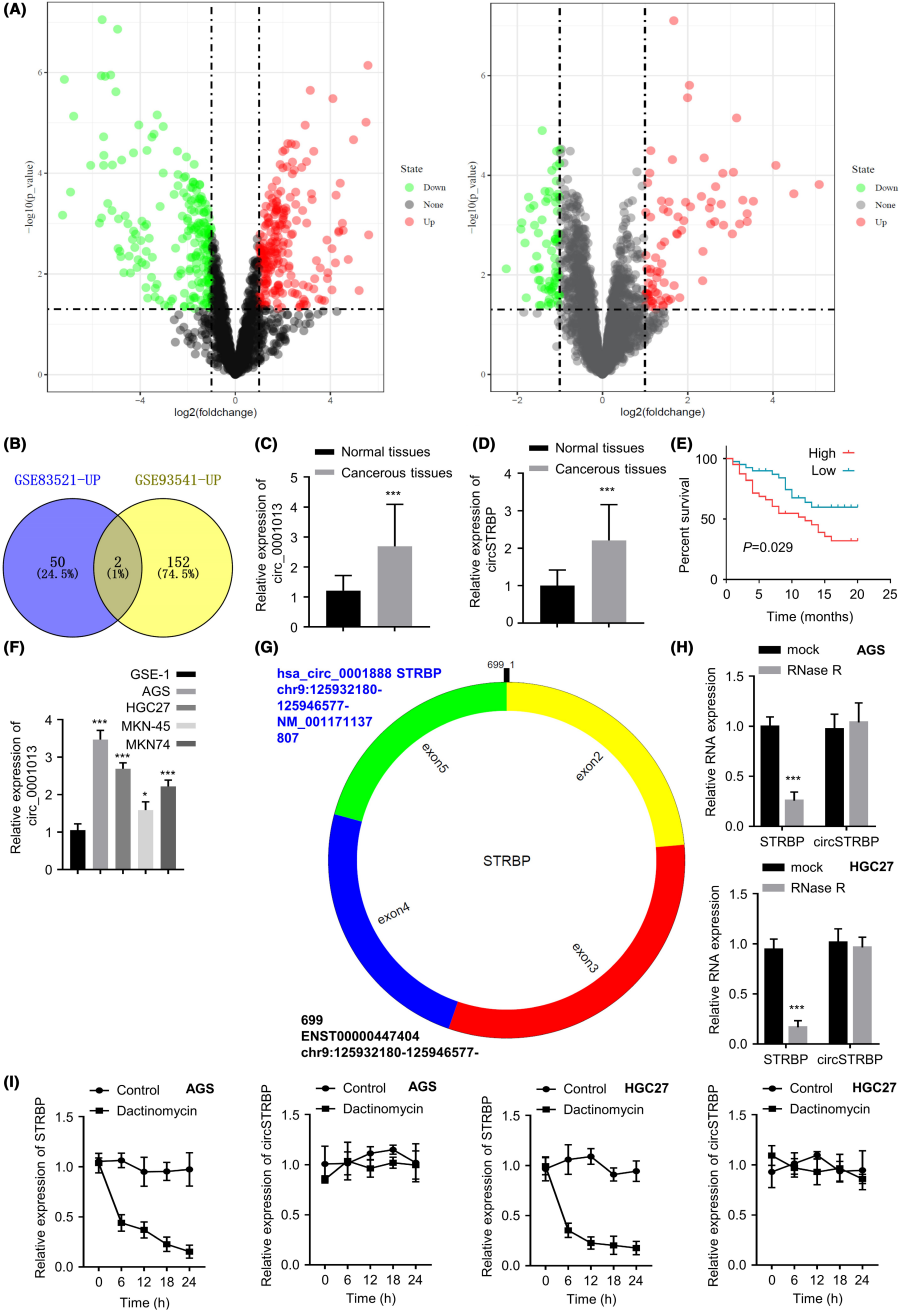
circSTRBP is highly expressed within GC cells and tissues. (A) Volcano plot showing the downregulated (in green) and upregulated (in red) circular RNAs in GC samples in comparison with normal tissues. The analyses are from GSE83521 and GSE93541 datasets. (B) The upregulated circRNA sets of GSE83521 and GSE93541 shared two common elements: hsa_circ_0001013 and circSTRBP. (C) circ_0001013 expression in 80 pairs of GC and para‐cancerous samples. (D) circSTRBP expression in 80 pairs of GC and para‐cancerous samples. (E) Overall survival of GC patients in circSTRBP high and low expression groups. (F) Relative expression of circSTRBP within GC cell lines (HGC27, AGS, MKN‐45 and MKN74) and normal gastric epithelial cells (GSE‐1). (G) Schematics of circSTRBP sequences and structures. (H) Relative RNA levels within HGC‐27 and AGS cells with/without RNase R exposure. (I) Relative RNA levels with or without actinomycin D treatment in AGS and HGC27 cells. *p < 0.05; ***p < 0.001.

We initially retrieved the circRNA profiling data of GC and normal samples from the public database (GSE83521 and GSE93541 microarray data). Gene expression analysis revealed the differentially expressed circRNAs in GC tissues compared with the normal specimens (Figure 1A). Among these circRNAs, more than 200 were upregulated (labelled as GSE83521‐UP and GSE93541‐UP, respectively) with the fold change >2 (*p* < 0.01). Hsa_circ_0001013 (hsa_circRNA_000684) and circSTRBP (hsa_circRNA_104,907/hsa_circ_0001888) are the two commonly upregulated circRNAs in the two datasets (Figure 1B). We also collected 80 clinical samples of GC tumours and adjacent normal tissues to examine hsa_circ_0001013 and circSTRBP expression. Both circ_0001013 and circSTRBP showed significant upregulation in GC tumour specimens (both *p* < 0.001; Figure 1C,D). Since the functional role of circ_0001013 has been characterized in GC by a recent study,^18^ we focused on the functional engagement of circSTRBP in GC progression in our study.

To determine the prognostic significance of circSTRBP in GC, we examined the clinicalpathological features of 80 GC cases and the associations with circSTRBP expression level (Table 1). The circSTRBP expression values in cancerous tissues were considered as binary variable (high or low), with the median value as threshold to classify the high‐ and low‐level groups (*n* = 40 cases in each category). The elevated circSTRBP level was related to more distant metastasis (DM) and advanced TNM stages (*p* < 0.05); however, there was no significant correlation to other factors (i.e., patient age, biological sex and tumour size). Kaplan–Meier curve further showed that the overall survival of GC patients was poorer in GC case with high level of circSTRBP expression (*p* = 0.029; Figure 1E).

**TABLE 1:** Correlations of circSTRBP expression with the clinicopathologic features in GC patients.

Characteristics	Number	Expression of circSTRBP		*p*‐Value
		Low	High	
Gender				
Male	53	29	24	0.2371
Female	27	11	16	
Age				
<60	27	15	12	0.4781
≥60	53	25	28	
Histological grade				
Low	36	22	14	0.0722
High/moderate	44	18	26	
Tumour invasion depth				
T1	10	2	8	0.0425
T1 + T2 + T3	70	38	32	
Lymph node invasion				
N0	25	14	11	0.4693
N1 + N2 + N3	55	26	29	
TNM stage				
I–II	44	27	17	0.0246
III–IV	36	13	23	
Distant metastasis				
M0	68	37	31	0.0603
M1	12	3	9	

Moreover, we examined circSTRBP levels within GC cells (HGC27, AGS, MKN74 and MKN‐45) and the human gastric epithelial GSE‐1 cells. circSTRBP levels were significantly higher in GC cell lines relative to the normal cells, with HGC27 and AGS showing the highest expression (*p* < 0.05 or *p* < 0.001; Figure 1F). As illustrated in Figure 1G, circSTRBP is produced by the back‐splicing of exons 2, 3, 4 and 5 from pre‐STRBP mRNA. To confirm the stability of circSTRBP, we carried out Ribonuclease R (RNase R) digestion as well as actinomycin D treatment. RNase R exposure significantly reduced STRBP mRNA level, whereas circSTRBP level remained unchanged (*p* < 0.001; Figure 1H). After the blocking of transcription by actinomycin D, STRBP mRNA level showed a significant decrease within 24 h, while no significant change was observed for circSTRBP expression (Figure 1I). The above results demonstrated that circSTRBP is substantially elevated within GC cells and tissues, which is related to the poor survival in GC patients.

### 3.2 circSTRBP is packaged into exosomes from GC cells

**FIGURE 2:**
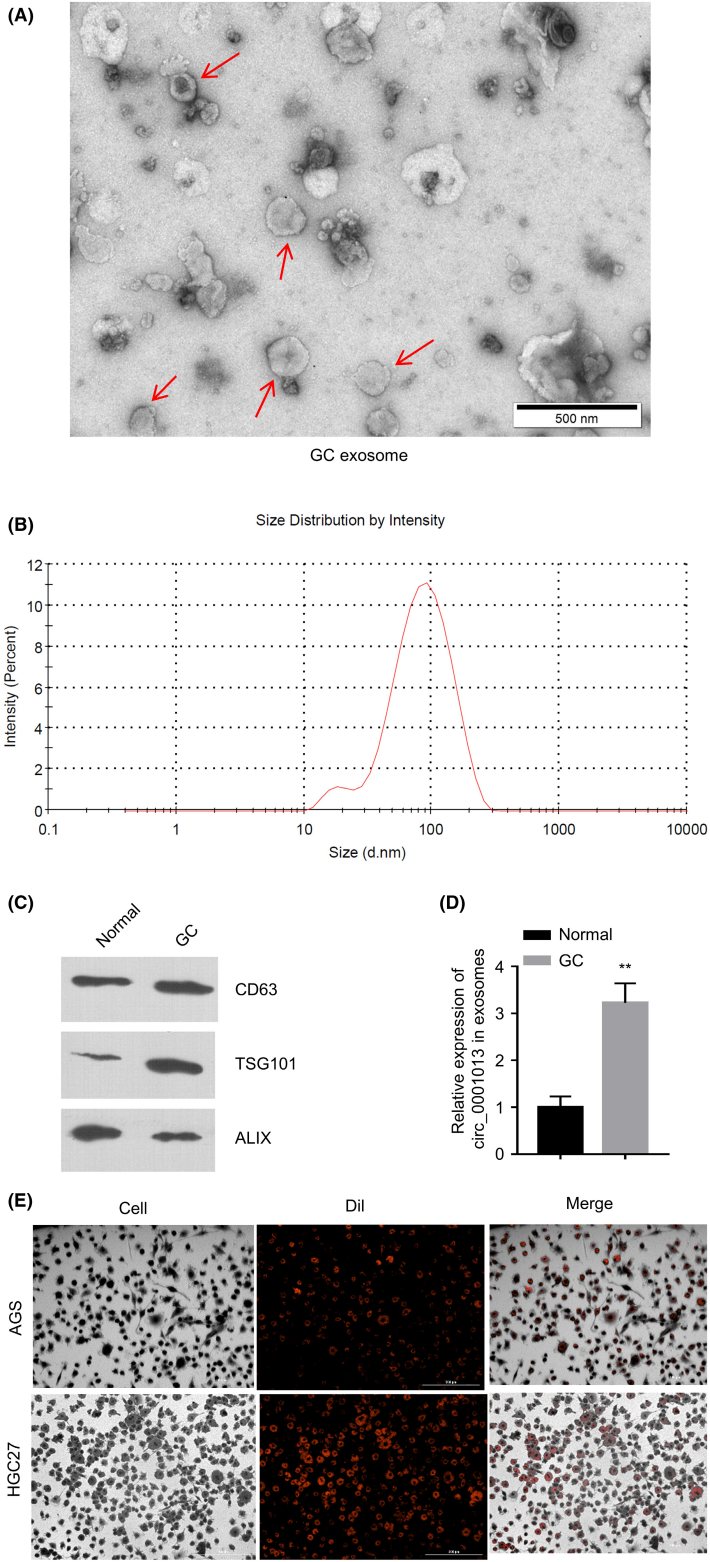
Package of circSTRBP in GC cell‐derived exosomes. (A) The transmission electron microscopy (TEM) image of GC cell (HGC27)–derived exosomes. Exosomes are marked by red arrows. (B) The size distribution of exosomes in the supernatant of GC cell culture. (C) Relative expression levels of CD63, TSG101 and ALIX in exosomal samples derived from normal gastric epithelial cells (GSE‐1) and GC cells (HGC27). (D) circSTRBP levels within the exosomes derived from normal gastric epithelial cells (GSE‐1) and GC cells (HGC27). (E) AGS and HGC‐27 cells were incubated with Dil‐labelled exosomes and the uptake of the exosomes was imaged under the fluorescence microscope. *p < 0.05; **p < 0.01; ***p < 0.001.

We next examined whether circSTRBP is secreted into the exosomes by GC cells (HGC27). Figure 2A shows the transmission electron microscopy (TEM) image of exosomal sample isolated from the supernatant of GC cell culture. Figure 2B displayed the size distribution of GC cell‐derived exosomes, which was determined by the nanoparticle tracking analysis (NTA). The WB assay for CD63, ALIX and TSG101 protein levels in exosomes derived from normal cells (GSE‐1) and GC cells (HGC27) showed that those exosomal markers were expressed in both exosomes (Figure 2C). RT‐qPCR quantification suggested that there was more abundant circSTRBP within the exosomes derived from GC cells compared with the normal cell‐derived exosomes (Figure 2D). We next incubated GC cells with Dil‐labelled exosomes isolated from GC cell culture, and the Dil‐labelled exosomes could be observed inside the two cell lines after incubation, as shown by phase‐contrast microscopy images (Figure 2E). Collectively, these data suggest that circSTRBP is secreted into GC cell‐derived exosomes.

### 3.3 Exosomal circSTRBP promotes GC cell growth and mobility

**FIGURE 3:**
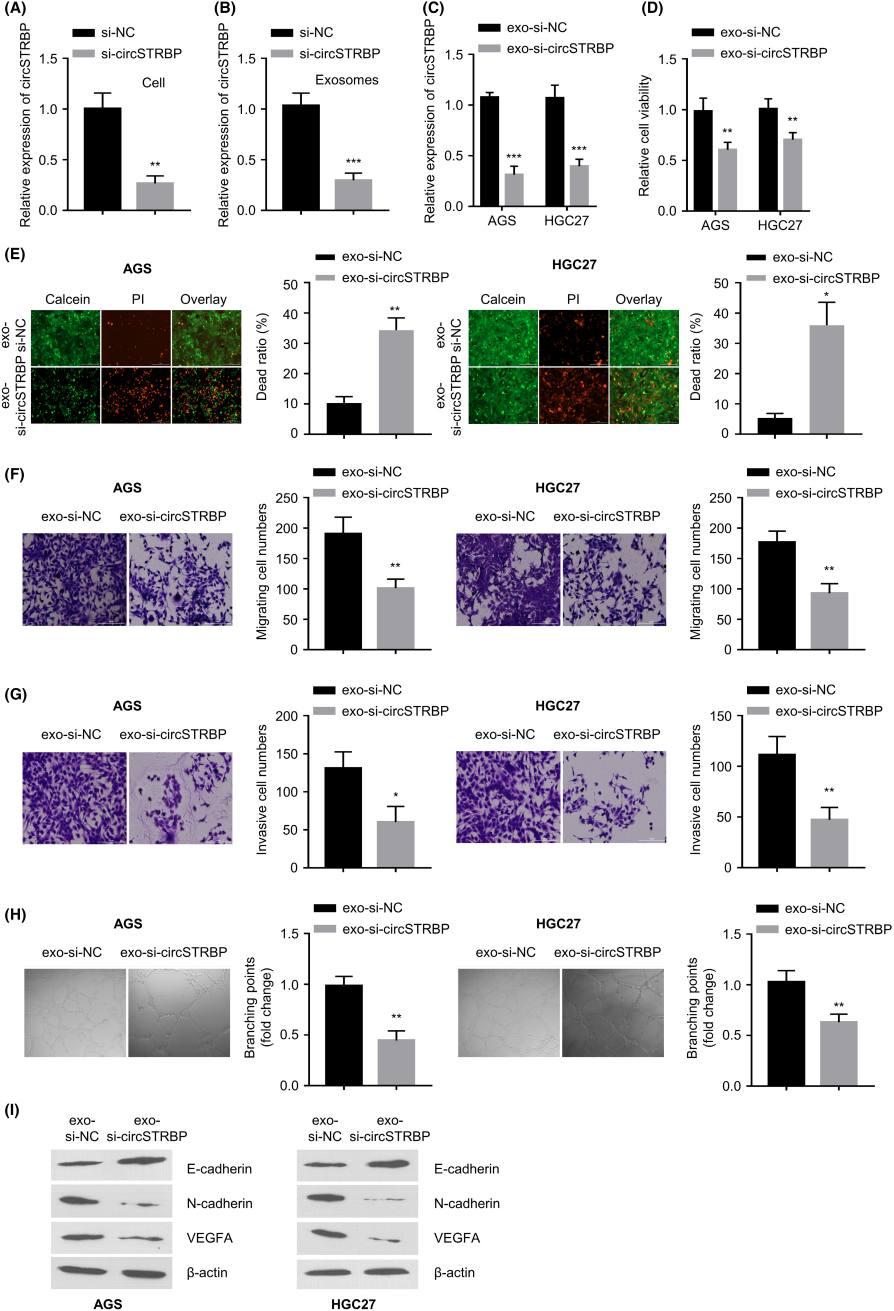
Exosomal circSTRBP promotes the survival and mobility of GC cells. (A) circSTRBP levels within HGC‐27 cells after the transfection of si‐NC (control siRNA) or si‐circSTRBP (siRNA targeting circSTRBP). (B) Relative expression levels of circSTRBP in exosomal samples isolated from HGC‐27 cells after the transfection of si‐NC or si‐circSTRBP. (C) Relative expression levels of circSTRBP within these two cell lines after the incubation with exosomal samples isolated from si‐circSTRBP‐treated (exo‐si‐circSTRBP) or si‐NC‐treated (exo‐si‐NC) HGC‐27 cells. (D) HGC‐27 and AGS cell viability after the incubation of exo‐si‐NC or exo‐si‐circSTRBP. (E) Calcein‐AM (green) and PI (red) staining in HGC‐27 and AGS cells after the incubation of exo‐si‐NC or exo‐si‐circSTRBP. (F) Transwell migration and (G) Invasion assays in HGC‐27 and AGS cells after the incubation of exo‐si‐NC or exo‐si‐circSTRBP. (H) Tube formation capacity in HGC‐27 and AGS cells after the incubation of exo‐si‐NC or exo‐si‐circSTRBP. (I) N‐cadherin, E‐cadherin, β‐Actin and VEGFA protein levels in HGC‐27 and AGS cells after the incubation of exo‐si‐NC or exo‐si‐circSTRBP. *p < 0.05; **p < 0.01; ***p < 0.001.

We next investigated the role of exosomal circSTRBP in GC cells by knockdown experiments. circSTRBP level was repressed after si‐circSTRBP (siRNA targeting circSTRBP) transfection in HGC27 cells when compared to the cells with si‐NC transfection (control siRNA) (*p* < 0.01; Figure 3A). Similarly, in the exosomal samples derived from GC cells with si‐circSTRBP transfection, circSTRBP expression level was significantly reduced (*p* < 0.001; Figure 3B). Moreover, we incubated AGS and HGC‐27 cells with exosomal samples isolated from si‐circSTRBP‐treated (exo‐si‐circSTRBP) or si‐NC‐treated (exo‐si‐NC) HGC‐27 cells. In both cells, circSTRBP expression level was significantly reduced after the incubation with exo‐si‐circSTRBP sample compared with exo‐si‐NC sample (*p* < 0.001; Figure 3C). As revealed by CCK‐8 assay, exo‐si‐circSTRBP group showed a decreased cell viability compared with exo‐si‐NC group (*p* < 0.01; Figure 3D). The analysis of cell viability and death by calcein‐AM/PI staining showed that the number of calcein‐AM‐positive cells (live) decreased and PI+positive (dead) cells increased in the exo‐si‐circSTRBP group (*p* < 0.01; Figure 3E). Transwell assays showed that incubation with exo‐si‐circSTRBP impaired the cell invasion and migration in both AGS and HGC‐27 cells (*p* < 0.01; Figure 3F,G). Furthermore, we evaluated the angiogenic potential and found that exo‐si‐circSTRBP group displayed an impaired tube formation capacity compared with exo‐si‐NC group (*p* < 0.01; Figure 3H). WB analysis also revealed that the incubation with exo‐si‐circSTRBP increased E‐cadherin level (epithelial marker), whereas the expression levels of angiogenic markers (VEGFA and N‐cadherin) were repressed (Figure 3I).

### 3.4 circSTRBP targets miR‐1294 and miR‐593‐3p

**FIGURE 4:**
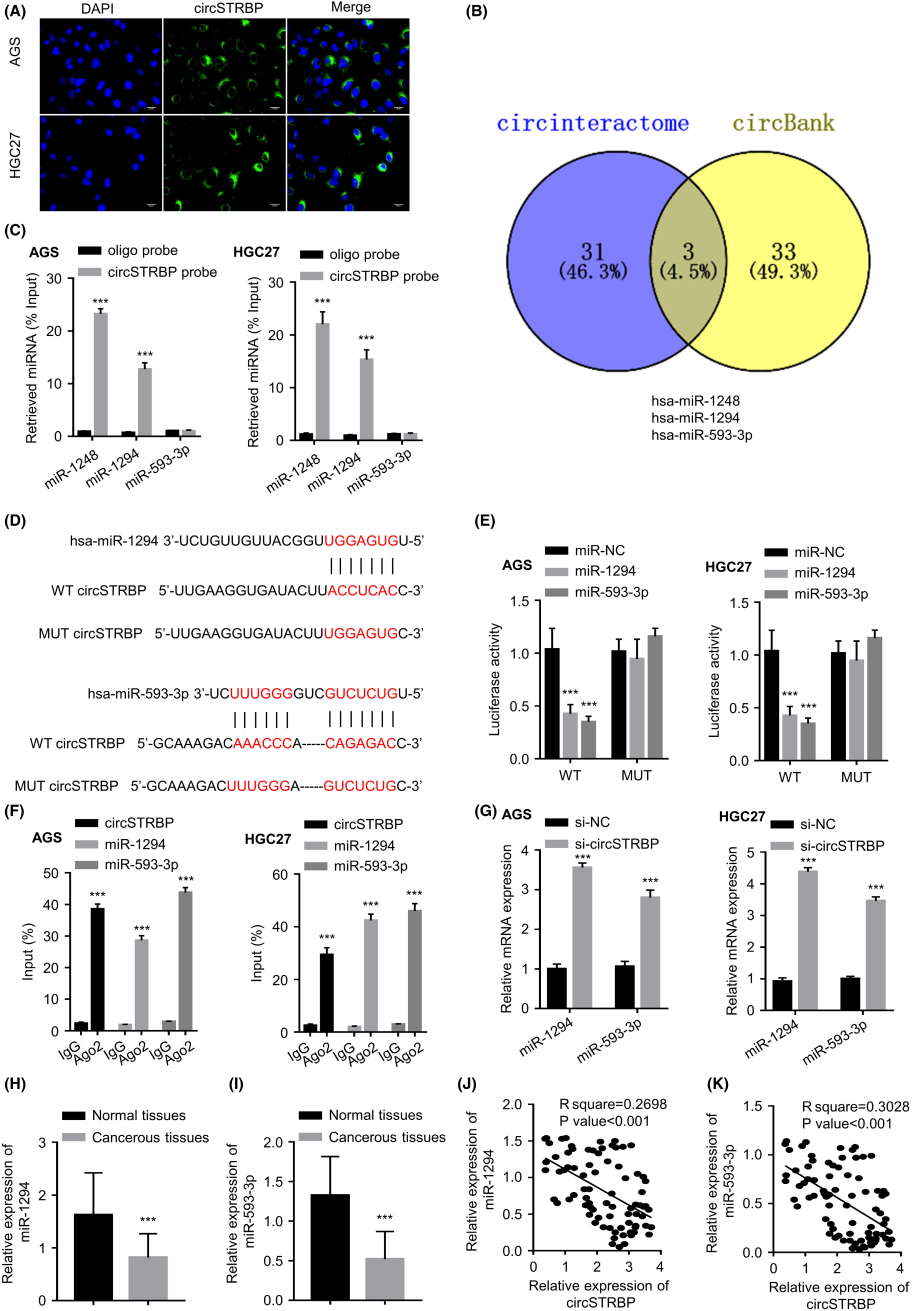
circSTRBP negatively targets miR‐1294 and miR‐593‐3p. (A) Subcellular location of circSTRBP in AGS and HGC‐27 cells by FISH staining. (B) Predicted miRNA targets of circSTRBP by two databases. (C) RNA pull‐down analysis by biotin‐labelled circSTRBP probe and control probe. (D) The predicted binding sites between circSTRBP and hsa‐miR‐593‐3p and hsa‐miR‐1294, respectively. (E) Dual luciferase activity assay in AGS and HGC‐27 cells (wide type or mutant luciferase reporter) when miR‐1294 or miR‐593‐3p was overexpressed. (F) Anti‐Ago2 RIP analysis of circSTRBP, miR‐593‐3p and miR‐1294 levels. (G) Relative RNA expression of miR‐1294 and miR‐593‐3p after circSTRBP knockdown. (H) miR‐1294 expression in 80 pairs of GC and matched normal tissues. (I) miR‐593‐3p expression in 80 pairs of GC and matched normal tissues. (J) Association of circSTRBP expression with miR‐1294 level in the GC tumour tissues. (K) Association of circSTRBP expression with miR‐593‐3p level in the GC tumour tissues. ***p < 0.001.

According to the fluorescence in situ hybridization (FISH) assay, circSTRBP showed a predominant cytoplasmic location in HGC‐27 and AGS cells (Figure 4A). CircInteractome (https://circinteractome.nia.nih.gov/) and circBank (http://www.circbank.cn/) online tools were employed to predict potential miRNA‐circRNA interactions for circSTRBP, and we identified three common candidate miRNAs from the two databases: hsa‐miR‐1248, hsa‐miR‐1294 and hsa‐miR‐593‐3p (Figure 4B). qRT‐PCR analysis showed that circSTRBP probe enriched both hsa‐miR‐1294 and hsa‐miR‐593‐3p, but not hsa‐miR‐1248 (*p* < 0.001; Figure 4C). Figure 4D illustrates the predicted binding sites in circSTRBP for hsa‐miR‐593‐3p and hsa‐miR‐1294, respectively. Compared with miR‐NC, miR‐593‐3p or miR‐1294 overexpression inhibited wild‐type (WT) circSTRBP luciferase reporter activity in HGC‐27 and AGS cells. Such inhibition was not observed when miR‐1294 or miR‐593‐3p's binding sites were mutated, suggesting the associations between circSTRBP and the miRNAs via predicted interacting sequences (*p* < 0.001; Figure 4E). We next performed an RNA immunoprecipitation (RIP) assay using IgG control and argonaute 2 (Ago2) antibody. Ago2 could precipitate circSTRBP, miR‐1294 and miR‐593‐3p to a much higher extent when compared to the IgG control (*p* < 0.001; Figure 4F). Furthermore, the knockdown of circSTRBP caused the increased miR‐1294 and miR‐593‐3p expression within the 2 GC cell lines (*p* < 0.01; Figure 4G). In the 80 pairs of GC tumours and matched non‐carcinoma sample, GC specimens displayed significantly lower levels of miR‐1294 and miR‐593‐3p (*p* < 0.001; Figure 4H,I). Besides, circSTRBP level showed a negative association with both miR‐1294 and miR‐593‐3p levels in the GC samples (*p* < 0.001; Figure 4J,K). Collectively, these findings indicate that circSTRBP interacts with miR‐1294 and miR‐593‐3p.

### 3.5 Exosomal circSTRBP regulates GC progression by targeting miR‐1294 and miR‐593‐3p

**FIGURE 5:**
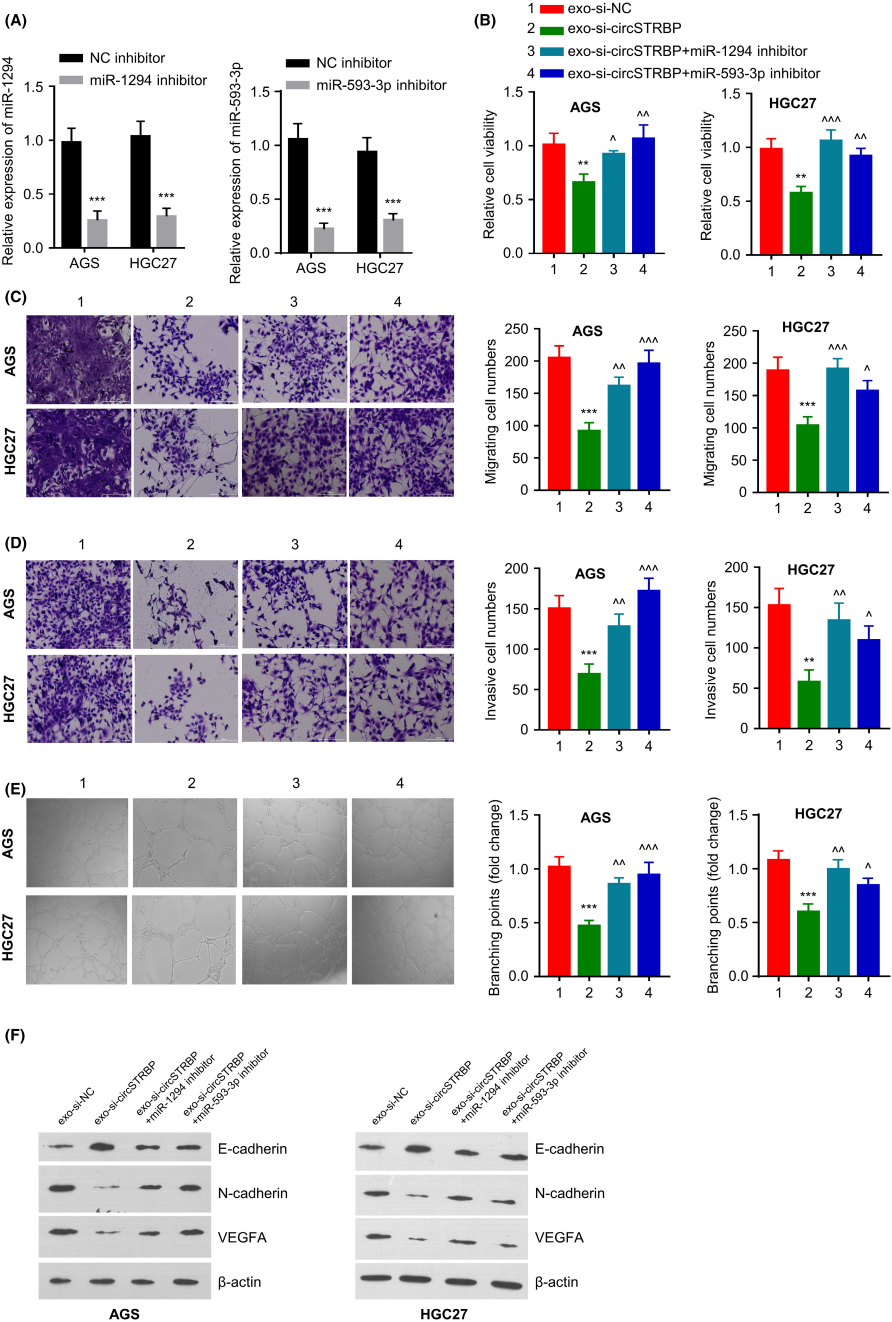
Exosomal circSTRBP regulates GC progression by targeting miR‐1294 and miR‐593‐3p. (A) Relative expression of miR‐1294 or miR‐593‐3p within HGC‐27 and AGS cells after the transfection of miR‐593‐3p or miR‐1294 inhibitor. (B–F). HGC‐27 and AGS cell were divided into exo‐si‐circSTRBP, exo‐si‐NC, exo‐si‐circSTRBP+miR‐593‐3p inhibitor or exo‐si‐circSTRBP+miR‐1294 inhibitor group. (B). CCK‐8 viability assay, (C) Transwell migration assay, (D) Transwell invasion assay, (E) Tube formation assay and (F) WB analysis of E‐cadherin, N‐cadherin, β‐Actin and VEGFA levels in each experimental condition. **p < 0.01; ***p < 0.001 versus exo‐si‐NC. ^^^ *p* < 0.05; ^^^^ *p* < 0.01; ^^^^^ *p* < 0.001 versus exo‐si‐circSTRBP.

We then studied whether miR‐1294 and miR‐593‐3p are implicated in the role of circSTRBP in GC cells. Within HGC‐27 and AGS cells, miR‐593‐3p or miR‐1294 inhibitor transfection could reduce miR‐593‐3p and miR‐1294 levels, respectively (*p* < 0.01; Figure 5A). The above two cell lines were incubated with exo‐si‐circSTRB, which suppressed the viability when compared to the exo‐si‐NC group (*p* < 0.01; Figure 5B). The co‐transfection of miR‐1294 or miR‐593‐3p inhibitor was able to rescue the cell viability in the exo‐si‐circSTRBP group (Figure 5B). Transwell assays revealed the reduced numbers of migrative and invasive cells in the exo‐si‐circSTRBP group, and the migratory and invasive abilities were enhanced in the presence of miR‐1294 inhibitor or miR‐593‐3p inhibitor (both *p* < 0.01; Figure 5C,D). The exo‐si‐circSTRBP group displayed an impaired tube formation capacity, while the application of miRNA inhibitor also promoted the tube formation (*p* < 0.01; Figure 5E). Moreover, in the exo‐si‐circSTRBP group, the decreased expression of N‐cadherin/VEGFA and the increased E‐cadherin level were attenuated by miR‐1294 and miR‐593‐3p inhibitors (Figure 5F). Altogether, exosomal circSTRBP exerts the effect on GC progression via interacting with miR‐1294 and miR‐593‐3p.

### 3.6 miR‐1294 and miR‐593‐3p could target E2F2

**FIGURE 6:**
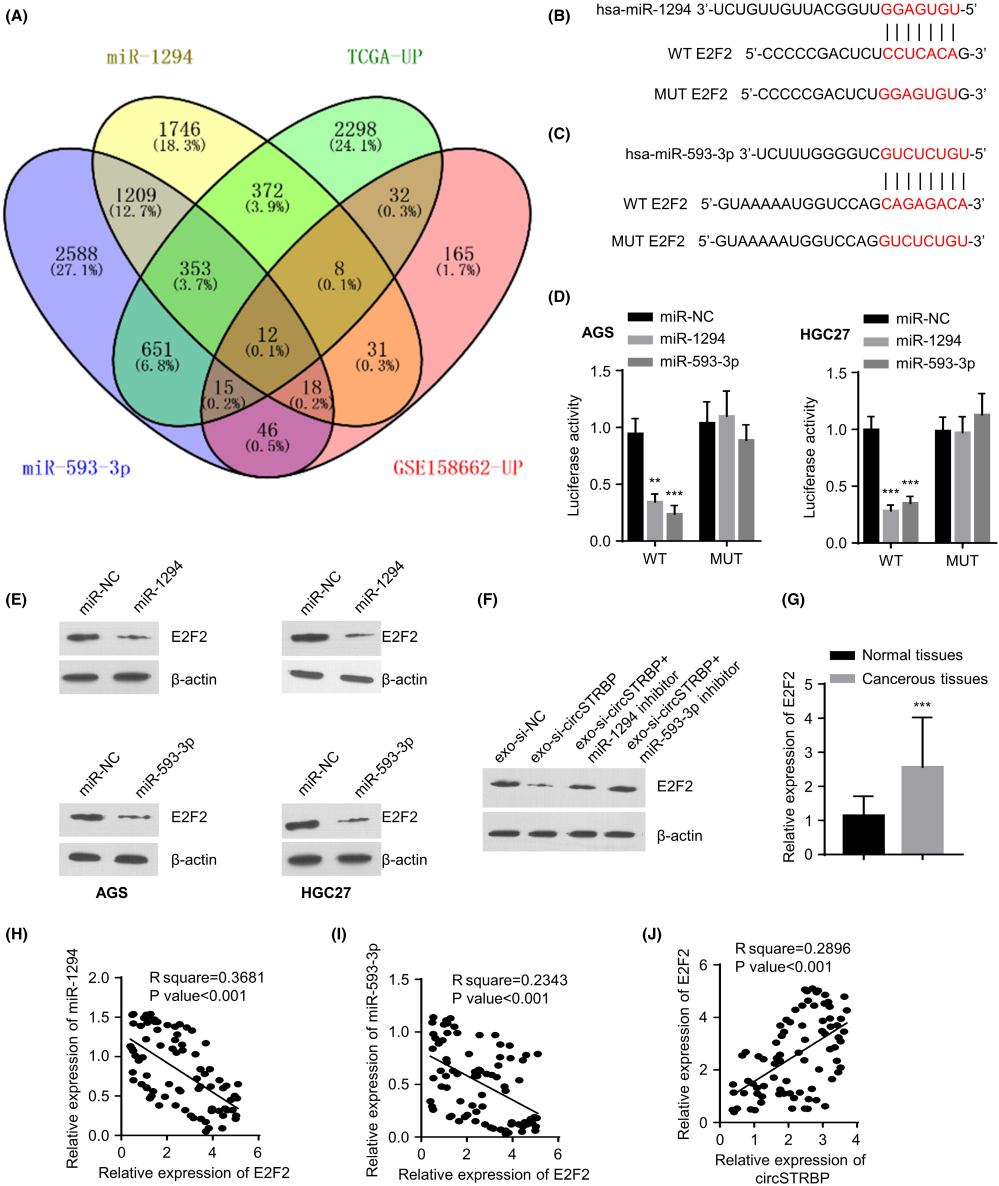
miR‐1294 and miR‐593‐3p negatively regulates E2F2 expression. (A) mRNA target prediction for miR‐1294 and miR‐593‐3p. (B) The predicted binding sites between E2F2 mRNA and miR‐1294. (C) The predicted binding sites between E2F2 mRNA and miR‐593‐3p. (D) Dual luciferase activity assay using the WT E2F2–3′‐UTR or MUT reporter after the transfection of miR‐NC, miR‐1294 or miR‐593‐3p mimics. (E) WB analysis of E2F2 and β‐Actin within HGC‐27 and AGS cells upon the transfection of miR‐NC, miR‐593‐3p or miR‐1294 mimics. (F) WB analysis of E2F2 and β‐Actin in HGC‐27 and AGS cells in exo‐si‐NC, exo‐si‐circSTRBP, exo‐si‐circSTRBP+miR‐1294 inhibitor or exo‐si‐circSTRBP+miR‐593‐3p inhibitor groups. (G) Relative E2F2 mRNA levels in GC tumours and the matched normal tissues. (H) Association of E2F2 mRNA level and miR‐1294 level in GC tumour specimens. (I) Association of E2F2 mRNA level with miR‐593‐3p level in GC tumour specimens. (J) Association of circSTRBP expression level with E2F2 mRNA level in GC tumour specimens. **p < 0.01; ***p < 0.001.

Targetscan database prediction of the potential targets of miR‐1294/miR‐593‐3p and the analyses with the genes upregulated in GC using the public datasets identified E2F2 as the common target of the two miRNAs (Figure 6A). Figure 6B,C show the binding sites predicted between E2F2 mRNA and miR‐593‐3p/miR‐1294, respectively. AGS and HGC‐27 cells were transfected with luciferase reporter vectors that contain the WT or MUT sequence of E2F2 mRNA. miR‐1294 and miR‐593‐3p mimics markedly reduced luciferase activities of WT E2F2 reporter, whereas no inhibition was seen in cells transfected with the MUT reporter (*p* < 0.01, *p* < 0.001, separately; Figure 6D). Additionally, both miR‐593‐3p miR‐1294 overexpression decreased E2F2 protein levels within these two cell lines (*p* < 0.01; Figure 6E). The exo‐si‐circSTRBP was able to suppress E2F2 protein level, which was antagonized by miR‐593‐3p and miR‐1294 inhibitor (Figure 6F). In the 80 pairs of GC tumour and normal specimens, E2F2 mRNA showed an upregulation in the cancerous tissues (*p* < 0.001; Figure 6G). Pearson correlation analyses further showed that E2F2 level was negatively related to the expression of miR‐593‐3p or miR‐1294 (both *p* < 0.001; Figure 6H,I). In contrast, E2F2 level displayed a positive association with circSTRBP level (*p* < 0.001; Figure 6J).

### 3.7 Exosomal circSTRBP promotes GC progression via modulating E2F2

**FIGURE 7:**
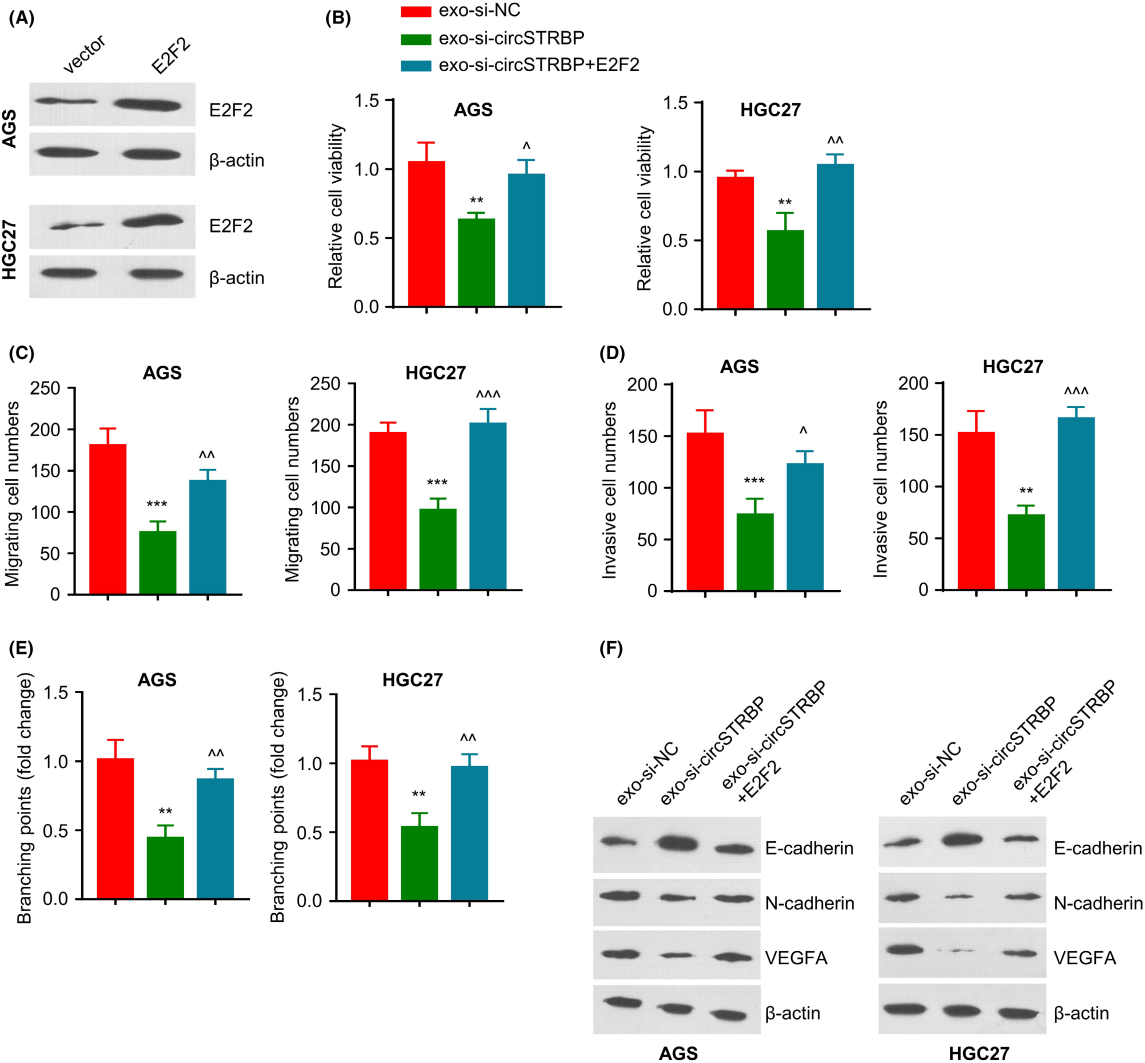
Exosomal circSTRBP promotes GC progression via modulating E2F2. (A) E2F2 levels within HGC‐27 and AGS cells after the transfection of empty vector or E2F2 expression vector. (B–F) HGC‐27 and AGS were divided into exo‐si‐NC, exo‐si‐circSTRBP or exo‐si‐circSTRBP+E2F2 transfection groups. (B). CCK‐8 viability assay, (C) Transwell migration assay, (D) Transwell invasion assay, (E) Tube formation assay and (F) WB analysis of E‐cadherin, N‐cadherin, β‐Actin and VEGFA levels in each experimental condition. **p < 0.01; ***p < 0.001 versus exo‐si‐NC. ^^^ *p* < 0.05; ^^^^ *p* < 0.01; ^^^^^ *p* < 0.001 versus exo‐si‐circSTRBP.

Next, we applied E2F2 expression vector to ectopically express E2F2 in AGS and HGC‐27 cells (*p* < 0.01; Figure 7A). The two cell lines were incubated with exo‐si‐NC, exo‐si‐circSTRBP or exo‐si‐circSTRBP+E2F2 expression vector. E2F2 overexpression promoted cell viability upon exo‐si‐circSTRBP incubation (*p* < 0.01; Figure 7B). Transwell experiments further demonstrated that E2F2 overexpression could rescue the cell migration and invasion in the cells upon exo‐si‐circSTRBP treatment (both *p* < 0.01; Figure 7C,D). Besides, the impaired tube formation ability after exo‐si‐circSTRBP incubation was also enhanced with E2F2 overexpression (*p* < 0.01; Figure 7E). exo‐si‐circSTRBP incubation reduced VEGFA and N‐cadherin levels and increased E‐cadherin expression, and these effects were largely abrogated upon E2F2 overexpression. (Figure 7F). These results suggest that exosomal circSTRBP modulates the aggressiveness of GC cells by targeting E2F2.

### 3.8 Exosomal circSTRBP promotes the tumorigenesis of GC cells in vivo

**FIGURE 8:**
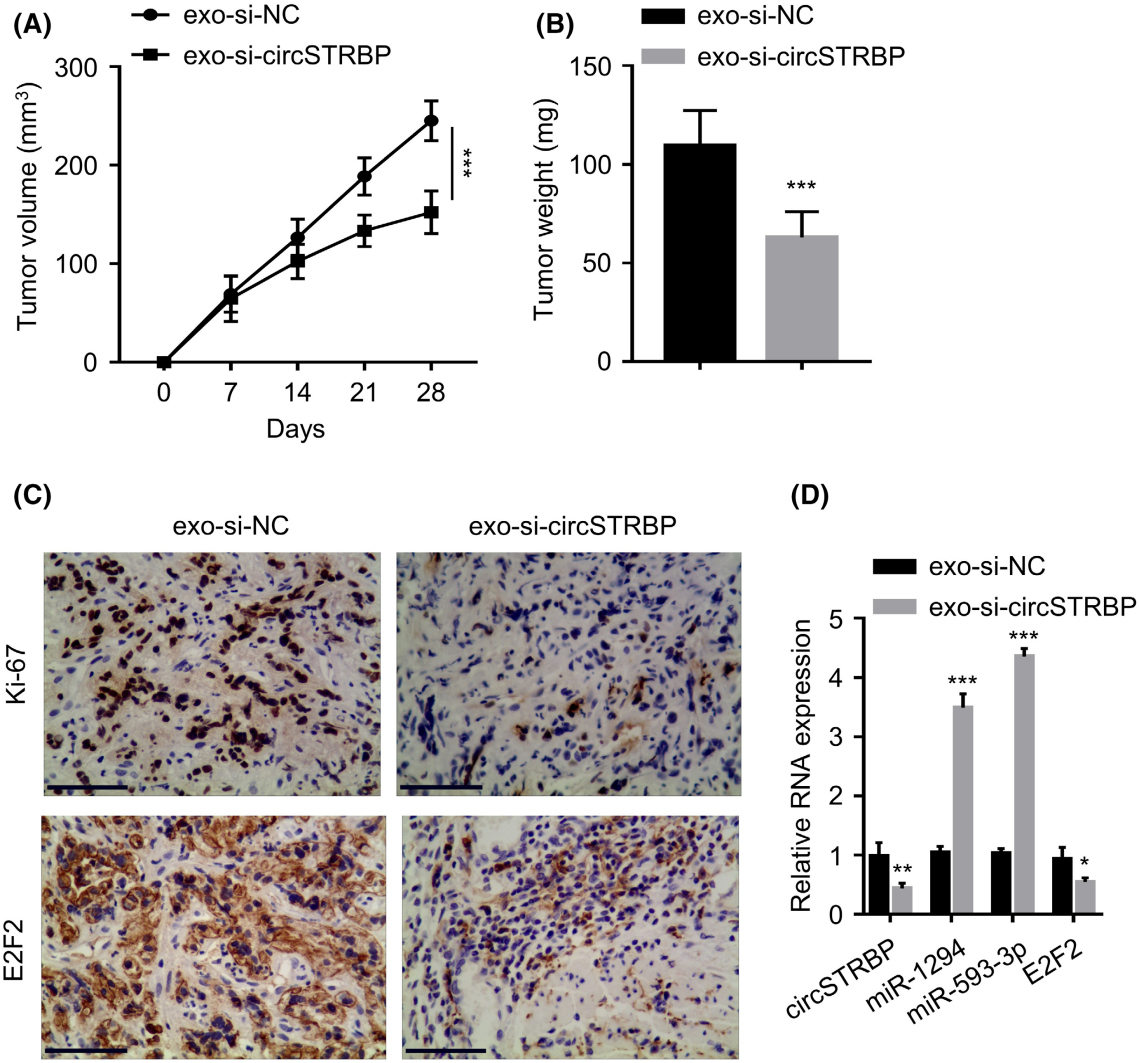
Exosomal circSTRBP promotes GC cell growth in vivo. (A and B) The volume (mm^3^) and weight (mg) of the xenograft tumours in exo‐si‐NC and exo‐si‐circSTRBP groups. (C) IHC staining of Ki‐67 and E2F2 in the xenograft tumour sections. (D) Relative RNA expression of circSTRBP, miR‐1294, miR‐593‐3p and E2F2 in tumour samples of exo‐si‐NC or exo‐si‐circSTRBP groups. *p < 0.05; **p < 0.01; ***p < 0.001.

To further demonstrate the pro‐tumorigenic effect of exosomal circSTRBP in vivo, nude mice were injected with AGS cells, and the mice were assigned into exo‐si‐circSTRBP group (intratumorally injected with exosomes extracted from circSTRBP siRNA‐transfected AGS cells) and exo‐si‐NC group (intratumorally injected with exosomes from AGS cells transfected with the control siRNA). The administration of exo‐si‐circSTRBP significantly attenuated the tumour formation of AGS cells in the nude mice (*p* < 0.05; Figure 8A,B). Besides, IHC staining of Ki‐67 and E2F2 in the xenograft tumours revealed that the reduction in E2F2 level in the exo‐si‐circSTRBP group was accompanied by the lowered level of Ki‐67 staining (*p* < 0.05; Figure 8C). We further examined the relative RNA levels of circSTRBP, miR‐1294, miR‐593‐3p and E2F2 in tumour tissues. Compared with the exo‐si‐NC samples, tumour tissues from the exo‐si‐circSTRBP group expressed lower levels of circSTRBP and E2F2 and the increased levels of miR‐1294 and miR‐593‐3p (*p* < 0.05; Figure 8D). These data further corroborate the oncogenic role of exosomal circSTRBP in promoting the tumorigenesis of GC cells in vivo.

## 4 DISCUSSION

GC is a common malignancy and a leading cause of cancer‐associated mortality globally.^1^ Understanding the molecular basis of GC progression is the key for early diagnosis and the formulation of novel treatment schemes. Emerging evidence pinpoint that the deregulation of circRNAs contributes to cancer development and progression. circRNAs have been characterized as a novel group of ncRNAs showing aberrant expression within several cancers such as GC.^19^ The work explored the functional engagement of exosomal circSTRBP derived from GC cells in dictating the aggressiveness and regulating the tumorigenesis of GC cells. According to our results, circSTRBP which was upregulated within GC cells and tissues was significantly enriched in GC cell‐derived exosomes. In addition, we provided evidence for functional roles of exosomal circSTRBP in promoting the cell viability, mobility and angiogenic potential in GC cells. Exosomal circSTRBP promotes the progression of GC through targeting miR‐1294 and miR‐593‐3p and modulating E2F2 expression. Exosomal circSTRBP also facilitates tumour growth in the mouse model of GC.

Previous evidence indicates that circRNAs are enriched in cancer cell‐derived exosomes.^20^, ^21^ These extracellular vesicles play pivotal roles in cellular communication by carrying a variety of cargo, including DNA, RNA, proteins and lipids, which could be taken up by the neighbouring cells to impact the functional properties.^20^ In cancers, exosomes can function to establish a favourable microenvironment for disease progression and metastases.^22^, ^23^ The study by Li et al.^24^ provided solid evidence supporting the enrichment of circRNAs within exosomes derived from cancer cells. In addition, exosomal circRNAs in the serum sample have been proposed to serve as biomarkers to discriminate colorectal cancer (CRC) cases from normal subjects, and there was an association between the expression levels of exosomal circRNAs and tumour mass.^24^ Following that, numerous studies have examined the association between exosomal circRNAs in serum and the clinical progressions in patients suffering hepatocellular cancer (HCC), GC and pancreatic cancer. These evidence highlighted the correlation between exosomal circRNA abundance with patients' clinical stage and metastasis.^25^, ^26^, ^27^, ^28^, ^29^, ^30^, ^31^ Our results added novel evidence regarding the overexpression of circSTRBP in GC tumours and characterized circSTRBP as a tumour‐promoting factor secreted in the exosomes by GC cells. Our data also indicated that circSTRBP may be employed as a prognostic biomarker for GC patients as its high expression level was associated with a poorer overall survival. Currently, there is no reports regarding the functional role of circSTRBP in other types of malignancies. Whether the exosomal circSTRBP could serve as a biomarker for GC diagnosis and prognosis needs to be validated in a large cohort of clinical samples. Furthermore, whether exosomal circSTRBP can shape the tumour microenvironment in GC warrants future investigation.

The most well‐established function of circRNAs is to serve as molecular sponge to limit the activity of miRNA targets. CDR1as, the first circRNA with characterized biological activity within exosomes, was found to act as a sponge of exosomal miR‐7.^24^ This study suggests that circRNAs in the exosomes preserve the activity to sponge miRNA and regulate the expression of downstream mRNA targets in the recipient cells. Notably, a single circRNA may contain multiple seeding sequences for adsorbing distinct miRNAs, which may function as a tumour‐suppressive or an oncogenic factor in different scenarios.^32^ According to our results, exosomal circSTRBP plays an oncogenic role via inhibiting miR‐1294 and miR‐593‐3p in GC cells. Previous studies showed that miR‐1294 is downregulated in GC and its reduced level is associated with the dismal survival in GC patients, supporting a tumour‐suppressive role of this miRNA.^14^, ^15^, ^16^ In agreement with these studies, we showed that miR‐593‐3p and miR‐1294 suppressed GC viability and aggressiveness. Furthermore, we showed that the two miRNAs target E2F2, a transcription regulator whose dysregulation can lead to aberrant cell proliferation and cell cycle progression.^33^ E2F2 was previously reported to be overexpressed in GC and miR‐31 was found as a regulator of E2F2 within GC cells. E2F2 silencing impaired the cell growth, invasion and migration of GC cells.^34^ Thus, our data are consistent with the reported pro‐tumorigenic role of E2F2 and provide novel insights into the regulation of E2F2 expression by circSTRBP/miR‐593‐3p/miR‐1294 axis in GC.

To conclude, our data revealed that circSTRBP exhibited a heightened expression within GC cells and tissues, and circSTRBP is enriched in exosomes derived from GC cells. Exosomal circSTRBP may promote the viability and the aggressiveness of GC cells via miR‐1294/miR‐593‐3p/E2F2 axis. Nevertheless, the clinical potential of circSTRBP as a circulatory biomarker for GC diagnosis requires further clarification. More investigations are needed to unravel the mechanisms underlying the packaging and trafficking of exosomal circSTRBP, as well as the biological functions in the tumour microenvironment. Further, future efforts are required to optimize the delivery method of circSTRBP targeting siRNA in animal models in order to evaluate the effect of circSTRBP silencing on GC progression.

## AUTHOR CONTRIBUTIONS

**Yin Wang:** Data curation (equal); formal analysis (equal); investigation (equal); methodology (equal); resources (equal); software (equal); validation (equal); visualization (equal); writing – original draft (equal). **Deke Li:** Methodology (equal); writing – review and editing (equal). **Rong Zou:** Data curation (equal); formal analysis (equal); investigation (equal); methodology (equal); resources (equal); software (equal); validation (equal); visualization (equal); writing – original draft (equal). **Xiankui Gao:** Data curation (equal); formal analysis (equal); investigation (equal); methodology (equal). **Xingjun Lu:** Conceptualization (equal); funding acquisition (equal); project administration (equal); supervision (equal); writing – review and editing (equal).

## FUNDING INFORMATION

The study was funded by the 2021 Bozhou City major research and development project (bzzc2021023).

## CONFLICT OF INTEREST STATEMENT

All the authors have no conflict of interest for this study.

## Data Availability

The data in the current study are available from the corresponding author upon reasonable request.
